# Transcription-Based Amplified Colorimetric Thrombin Sensor Using Non-Crosslinking Aggregation of DNA-Modified Gold Nanoparticles

**DOI:** 10.3390/s21134318

**Published:** 2021-06-24

**Authors:** Yu Muto, Gen Hirao, Tamotsu Zako

**Affiliations:** 1 Department of Chemistry and Biology, Graduate School of Science and Engineering, Ehime University, 2-5 Bunkyo, Matsuyama 790-8577, Japan; yuu-mutou-qr@tosoh.co.jp (Y.M.); f310165u@mails.cc.ehime-u.ac.jp (G.H.); 2 Tokyo Research Center, TOSOH Corporation, 2743-1 Hayakawa, Ayase 252-1123, Japan

**Keywords:** aptamer, thrombin, gold nanoparticle, signal amplification, RNA polymerase

## Abstract

Gold nanoparticles (AuNPs) have been employed as colorimetric biosensors due to the color difference between their dispersed (red) and aggregated (blue) states. Although signal amplification reactions triggered by structural changes of the ligands on AuNPs have been widely used to improve measurement sensitivity, the use of ligands is limited. In this study, we designed a AuNP-based signal-amplifying sandwich biosensor, which does not require a conformational change in the ligands. Thrombin was used as a model target, which is recognized by two different probes. In the presence of the target, an extension reaction occurs as a result of hybridization of the two probes. Then RNA synthesis is started by RNA polymerase activation due to RNA promoter duplex formation. The amplified RNA drives aggregation or dispersion of the AuNPs, and a difference of the color if the AuNP solution is observed. As this detection system does not require a conformational change in the ligand, it can be generically applied to a wide range ligands.

## 1. Introduction

Gold nanoparticles (AuNPs) have recently been applied in various biosensors due to their unique electrochemical and optical properties [1,2,3,4]. In particular, in the construction of colorimetric biosensors, the color difference between aggregated and dispersed AuNPs has been utilized [5,6]. For example, the formation of AuNP aggregates can be easily recognized by the color change from red to blue/purple, and vice versa. A variety of biosensors using the color changes based on the aggregation or dispersion of AuNPs in the presence of analytes have been described [7,8]. AuNPs were also used for the detection of DNA, proteins, metal ions, and chemical compounds [9,10,11,12,13,14,15,16].

Because the color change of AuNPs is observed only when there is a significant difference in aggregation or dispersion [17], the use of aptamers as ligands has been combined with a signal amplification reaction triggered by their structural change to improve measurement sensitivity [18,19,20,21,22]. For example, Zou et al. proposed a signal-amplified color change assay that converts the conformational change of aptamers by target molecules into a hybridization chain reaction [19]. Chang et al. proposed a signal amplification-type colorimetric sensor that utilizes target recycling by an aptamer conformational change [20]. However, these aptamer conformational change-based systems cannot be applied to ligands such as antibodies and lectins, which are frequently used in the field of bioassays. In addition, it has been difficult to adapt the system to sandwich-type assays, which are widely used in the field of immunodiagnosis due to the high specificity obtained by dual recognition.

**Scheme 1:**
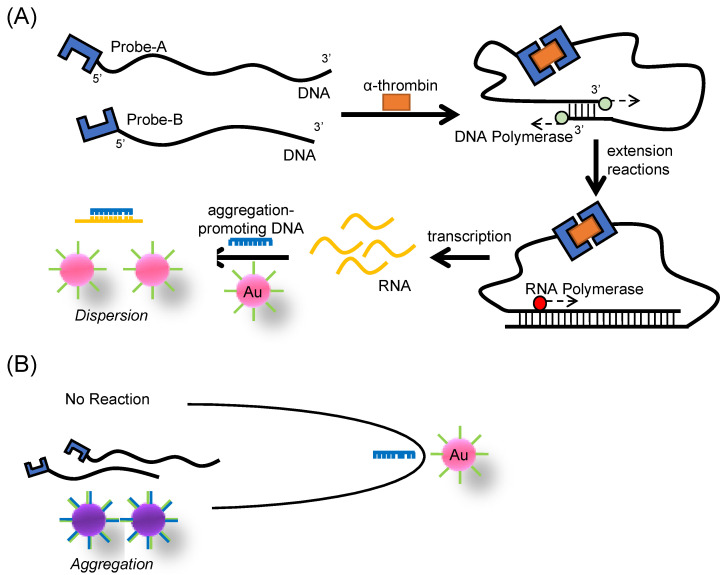
Schematic illustration of the transcription-based amplified colorimetric sensor in the (**A**) presence and (**B**) absence of a target molecules.

In this study, we designed a signal-amplified sandwich biosensor that does not require a conformational change in the ligand using the color differences of AuNP solutions. For signal amplification, we used the transcription reaction by the T7 RNA polymerase, which can perform a complete transcription cycle in the absence of additional protein factors [23,24] and has previously been used for molecular detection [25,26]. Thrombin was used as a model target protein that is recognized by two different probes [27]. The designed sensor involves two DNA probes that can specifically bind to thrombin. The binding of these probes leads to the hybridization of the 3′-end between the probes. This hybridization then triggers the extension reaction by a DNA polymerase to form a T7 RNA promoter duplex structure (Scheme 1). Therefore, T7 RNA polymerase can recognize the promoter duplex, and RNA strands are cyclically synthesized by the transcription reaction.

In this study, a colorimetric readout was achieved by utilizing the salt-induced non-crosslinking aggregation of AuNPs. This is based on the unique colloidal stability of AuNPs modified with DNA. The fully matched duplex formation on the AuNP surface triggers the rapid aggregation of the double-stranded (ds) DNA–AuNPs in a non-crosslinking manner at a high salt concentration, while single-strand DNA (ssDNA)-modified AuNPs can disperse owing to the interparticle electrostatic and steric repulsion generated by the surface-grafted ssDNA [28]. It was shown that the non-crosslinking AuNP aggregation can be controlled by the concentration of the fully matched ssDNA [20], which we named “aggregation-promoting DNA (agDNA)”. We speculated that we could control the amount of free agDNA by using RNA synthesized in the presence of the target molecule to trap the free agDNA. We can therefore detect the target molecule by analyzing the dispersion or aggregation state of AuNPs. AuNPs are dispersed in the presence of the target and are aggregated in its absence. We successfully demonstrated that thrombin can be detected with the described colorimetric sensor. As no structural changes in the ligands are necessary in this method, we suggest to apply it to various sandwich methods to detect the targets by color difference using signal amplification.

## 2. Materials and Methods

### 2.1. Reagents and Apparatus

AuNPs (20 and 40 nm) were obtained from BBI Solutions (Cardiff, UK). All oligonucleotides (Probe-A, Probe-B, ssDNA-15, ssDNA-20, agDNA-15, agDNA-20, RNA-15, NA, NB, and MB) shown in Figure S1 were purchased from FASMAC (Kanagawa, Japan). T7 RNA polymerase was purchased from Takara Bio Inc. (Shiga, Japan). 96-7 DNA polymerase and RNase inhibitor were purchased from Nippon Gene (Tokyo, Japan). Deoxynucleotide solution mix (dNTPs) and ribonucleotide solution mix (rNTPs) were purchased from New England Biolabs Inc. (Ipswich, ME, USA). Human α-thrombin was purchased from Funakoshi (Tokyo, Japan). Transferrin, human immunoglobulin G (IgG), human serum albumin (HSA), and bovine serum albumin (BSA) were purchased from Merck (Munich, Germany). NAP-5 columns were provided with the oligonucleotides. Absorption spectra and fluorescence intensities were measured using microplate reader (TECAN, Zürich, Switzerland). All photographs were taken using a digital camera (SONY, Tokyo, Japan).

### 2.2. Preparation of ssDNA-Modified AuNPs

ssDNA-modified AuNPs were prepared using thiolated ssDNA (ssDNA-15, ssDNA-20), as previously described [28]. Briefly, the thiolated DNA was incubated with 100 mM dithiothreitol (DTT), and the DTT was subsequently removed using a NAP-5 column. Thiolated ssDNA (3 nmol) was incubated with 1 mL of AuNP solution at 50 °C for 16 h. The solution was modified to NaCl (0.1 M) and phosphate buffer (10 mM, pH 7) by the addition of the necessary salts and was kept at 50 °C for 40 h. Excess DNA was removed by replacing the supernatant after centrifugation and redispersion in the same buffer. The washing process was repeated three times, and the precipitate was redispersed in 0.25 mL of the same buffer to make a stock solution that was used for the following experiments.

The amount of the modified ssDNA was estimated by a previously described method [29]. In brief, the prepared ssDNA-modified AuNP solution (200 μL) was centrifuged (14,000 and 9000 rpm for 20 and 40 nm AuNPs, respectively). The supernatant was removed, and 10% 2-mercaptoethanol (35 μL) was added to remove the ssDNA immobilized on AuNPs; the solution was incubated at 25 °C for 16 h and then centrifuged (25 °C, 14,000 rpm, 15 min). The amount of ssDNA on the AuNPs in the supernatant was quantified using the QuantiFluor ssDNA System (Promega, Madison, WI, USA). The amount of determined ssDNA determined for each AuNP was 280 ± 15 molecules for 20 nm AuNPs modified with 15-base ssDNA, 246 ± 12 molecules for 20 nm AuNPs modified with 20-base ssDNA, 485 ± 34 molecules for 40 nm AuNPs modified with 15-base ssDNA, and 475 ± 74 molecules for 40 nm AuNPs modified with 20-base ssDNA.

### 2.3. Color Change Performance of Each ssDNA-Modified AuNP Solution

Various concentrations of agDNA (agDNA-15, agDNA-20, 2.5 μL) were added to an aqueous solution of 1M phosphate buffer (pH 7.0, 5 μL), 20 M NaCl (2.5 μL), distilled water (32.5 μL), and the prepared ssDNA-modified AuNPs (7.5 μL); this was incubated for 15 min to induce AuNP aggregation. UV–vis spectra (400–800 nm) were measured using a microplate reader.

### 2.4. Colorimetric Detection of RNA Using ssDNA-Modified AuNPs

Various concentrations of RNA (RNA-15, 1 μL) were added to an aqueous solution (1 μL) of agDNA (agDNA-15) at each concentration (1, 2, 4 μM) and incubated at room temperature for 15 min. After this, 1 M phosphate buffer (pH 7.0, 2 μL), 20 M NaCl (1 μL), distilled water (12 μL), and the prepared ssDNA-modified AuNPs (3 μL) were added and incubated for 15 min to induce AuNP aggregation. UV–vis spectra (400–800 nm) were measured using a microplate reader. The degree of nanoparticle aggregation was evaluated by calculating the absorbance ratio (A_630_/A_530_).

### 2.5. Fluorescent Detection of Thrombin Using Molecular Beacons

A mixture of 0.6 μM thrombin (5 μL), 10 μM Probe-A (0.6 μL), 10 μM Probe-B (0.6 μL), 10 μM molecular beacon (MB, 0.9 μL), DMSO (4.5 μL), 10 × T7 RNA polymerase buffer (3 μL, 400 mM Tris-HCl, pH 8.0, 80 mM MgCl_2_, 20 mM spermidine), 100 mM DTT (1.5 μL), 25 mM NTP (3.6 μL), 10 mM dNTP (0.76 μL), 10 U/μL RNase inhibitor (0.6 μL), 8 U/μL 96-7 DNA polymerase (1 μL), 50 U/μL T7 RNA polymerase (2.88 μL), and distilled water (5.06 μL) was incubated at 42 °C for 30 min. The fluorescence intensity change was measured using a microplate reader with excitation at 470 nm and emission at 520 nm.

### 2.6. Colorimetric Detection of Thrombin Using ssDNA-Modified AuNPs

A mixture of various concentrations of thrombin (5 μL), 10 μM Probe-A (0.6 μL), 10 μM Probe-B (0.6 μL), DMSO (4.5 μL), 10 × T7 RNA polymerase buffer (3 μL), 100 mM DTT (1.5 μL), 25 mM NTP (3.6 μL), 10 mM dNTPs (0.76 μL), 10 U/μL RNase inhibitor (0.6 μL), 8 U/μL 96-7 DNA polymerase (1 μL), 50 U/μL T7 RNA polymerase (2.88 μL), and distilled water (5.96 μL) was incubated at 42 °C for 2 h to allow the transcription reaction. An aliquot (1 μL) of the mixture was added to an aqueous solution (1 μL) of a 1 μM agDNA-15 and incubated at room temperature for 15 min. Next, 1 M phosphate buffer (pH 7.0, 2 μL), 20 M NaCl (1μL), distilled water (12 μL), and prepared ssDNA-modified AuNPs (3 μL) were added and incubated for 15 min to induce AuNP aggregation. Pictures were taken with a digital camera, and UV–vis spectra (400–800 nm) were measured using a microplate reader. The degree of nanoparticle aggregation was evaluated by calculating the A_630_/A_530_ ratio.

## 3. Results and Discussion

### 3.1. Design of A Thrombin Detection System

In this study, we propose a signal-amplified sandwich-type sensor based on the color difference of AuNPs, which is independent of the conformational change in the ligand. Aptamers for thrombin were used as ligands for the construction of the sandwich-type assay system. It has been reported that aptamers for thrombin show high affinity for thrombin and that two aptamers can bind to a single molecule in a sandwich-type assay [27,30,31]. First, we designed a system of transcription-based amplified colorimetric sensors, as shown in Scheme 1. This system consisted of two DNA probes (Probe-A and Probe-B) (Figure S1), DNA polymerase, RNA polymerase, and ssDNA-modified AuNPs. Probe-A contains three segments: an antithrombin DNA aptamer (NU172) [30], T7 RNA promoter sequences, and the complementary sequence to the 3′-end segment of Probe-B. Probe-B contains two segments: an antithrombin DNA aptamer (HD22) [30] and a complementary sequence to the 3′-end segment of Probe-A.

The complementary sequence of each probe was set to six bases because this length did not lead to duplex formation in the absence of the target [32,33,34]. In the presence of thrombin (Scheme 1A), a sandwich-type interaction between thrombin and the DNA aptamers occurs, and DNA polymerase performs a DNA extension reaction from the 3′-end of the DNA where the duplex is formed. RNA polymerase then repeats RNA synthesis due to the presence of RNA promoter sequence duplexes. The synthesized RNA was utilized to modulate the AuNP aggregation process for colorimetric thrombin detection.

It has been reported that the aggregation of ssDNA-modified AuNPs is promoted by the double-strand formation between the surface ssDNA and the complementary strand [20,28]. In the proposed method, this complementary strand was used as agDNA that was trapped by RNA to inhibit the AuNP aggregation. As a result, no AuNP aggregation occurred in the presence of thrombin. In contrast, in the absence of thrombin (Scheme 1B), no RNA was synthesized because RNA promoter duplexes associated with the DNA extension reaction were not formed. agDNA, which is not trapped by RNA, then hybridizes with the ssDNA-modified on the AuNP surface to form the AuNP aggregation. As a result, the AuNP solution exhibited a purple color.

### 3.2. Effect of the AuNP Particle Size and ssDNA Length on AuNP Aggregation

**Figure 1:**
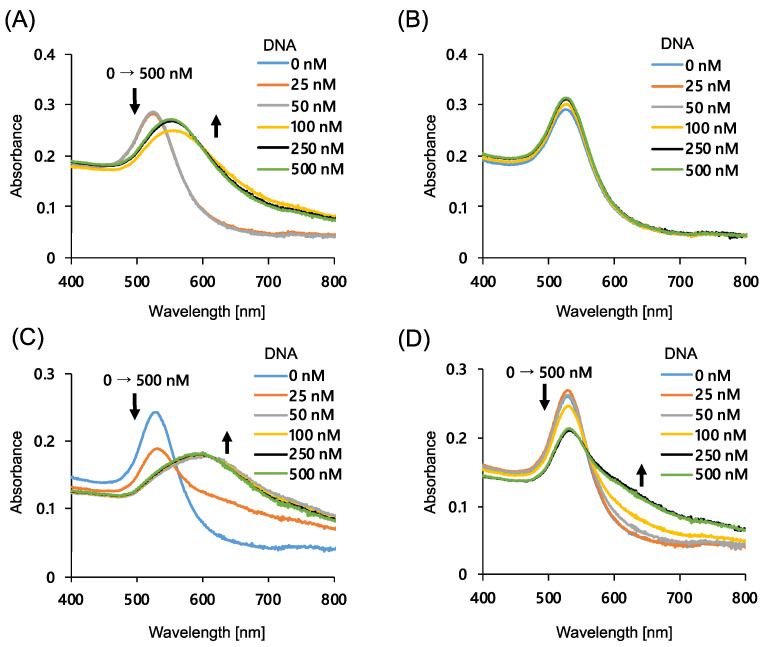
Absorption spectra change of AuNPs at different complementary DNA concentrations. (**A**) 20 nm AuNPs modified with 15-bases DNA; (**B**) 20 nm AuNPs modified with 20-bases DNA; (**C**) 40 nm AuNPs modified with 15-bases DNA, and (**D**) 40 nm AuNPs modified with 20-bases DNA.

First, we optimized the AuNP size and the chain length of the modified ssDNA to achieve a more distinct color change during AuNP aggregation. It has been reported that AuNPs modified with ssDNA rapidly aggregate in the presence of NaCl and complementary strands [28]. AuNPs with size of 20 and 40 nm were modified with 15- and 20-base thiolated ssDNA, and the degree of color change upon the addition of the complementary ssDNA was evaluated using absorption spectra (Figure 1). As shown in the figure, almost no spectral change was observed for 20 nm AuNPs modified with 20-base DNA (Figure 1B), and the largest spectral change was observed for 40 nm AuNPs modified with 15-base ssDNA (Figure 1C). This result can be attributed to two factors: the improved dispersion stability of the nanoparticles due to steric repulsion in the case of long DNA strand-modified nanoparticles [35], and the larger nanoparticles that are more likely to aggregate because of the difference in van der Waals forces depending on the size of the particles [36]. Thus, 40 nm AuNPs modified with 15-base DNA were used for the further study. It was also shown that the addition of more than 50 nM of complementary ssDNA was sufficient for the aggregation of the AuNPs under these conditions.

### 3.3. Modulation of AuNP Aggregation by RNA

Next, to confirm that RNA could be detected by the color difference of DNA-immobilized AuNPs, we checked whether the presence of RNA could prevent the aggregation of AuNPs by adding agDNA to 40 nm AuNPs modified with 15-base DNA. The characterization of the DNA-immobilized AuNPs was done by using dynamic light scattering (DLS) and TEM (Figure S2). The modification of ssDNA to the AuNPs was successfully confirmed by the increase in the particle size (Figure S2A). In addition, the ssDNA modification was also supported by the increase in negative charge in the zeta potentials measurement (Figure S2B). DLS measurement and TEM observation (Figure S2C) also supported the dispersed state of ssDNA-AuNPs.

**Figure 2:**
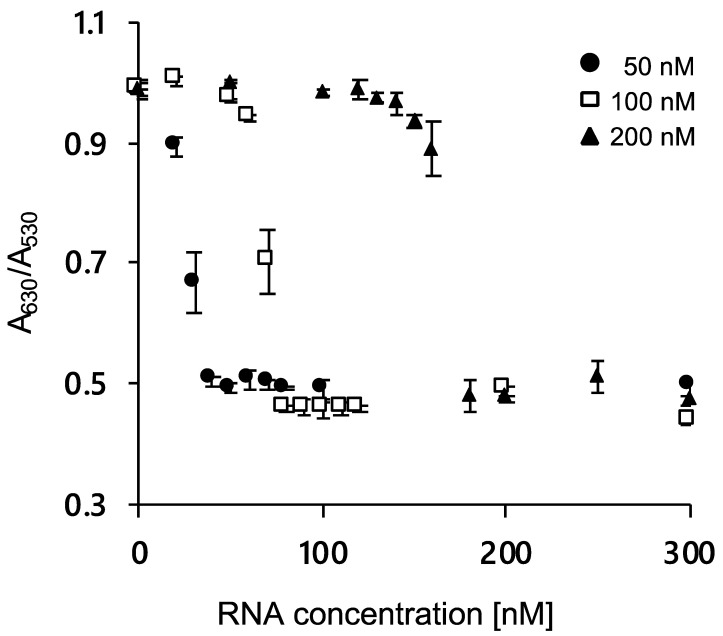
Ratio of absorbance at 630 and 530 nm of 40 nm AuNPs modified with 15-base DNA at various RNA concentrations. Presence in ●, 50 nM; □, 100 nM; and ▲, 200 nM agDNA-15. Error bars indicate the standard deviation of the three measurements.

The color difference due to AuNP aggregation was evaluated by measuring the absorbance ratio of A_630_/A_530_. Figure 2 shows the absorbance ratio after the addition of 0–300 nM RNA and agDNA (50, 100, and 200 nM). As the RNA concentration increased, the absorbance ratio decreased, indicating that the addition of RNA could inhibit nanoparticle aggregation. We also confirmed that the amount of RNA required for aggregation inhibition increased with the increasing concentrations of agDNA. These results showed that RNA could be detected by the color difference of DNA-immobilized AuNPs, supporting the feasibility of our system.

We also evaluated whether RNA could be synthesized by the system shown in Scheme 1 using the molecular beacon technique. The addition of thrombin amplified the fluorescent signal, indicating that RNA amplification has been triggered (Figure S3). Further, when an enzyme or aptamer site was missing, the fluorescent signal was not amplified by the addition of thrombin (Figure S3), indicating that both enzyme and aptamer sites are essential in this system. Additionally, the RNA amplification happens due to the formation of an RNA promoter duplex following DNA extension triggered by the addition of thrombin.

### 3.4. Thrombin Detection Using the Color Difference of AuNPs

**Figure 3:**
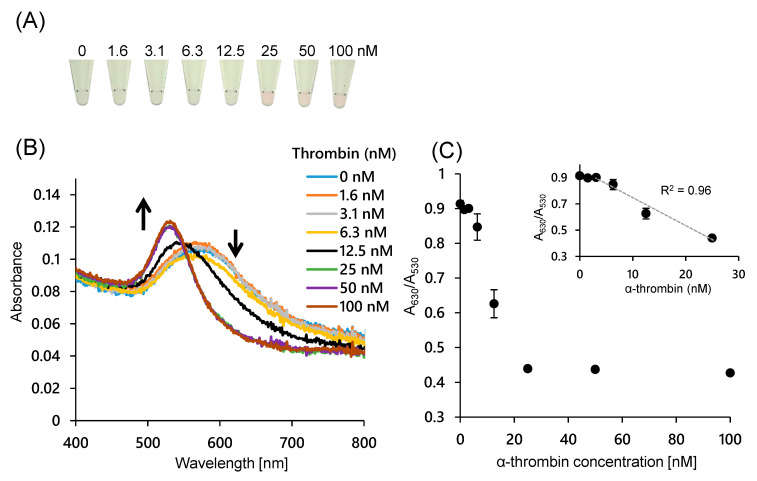
Analytical performance of the colorimetric thrombin assay using 40 nm AuNPs modified with 15-bases DNA. Thrombin concentration indicates the concentration during the transcription reaction. (**A**) Photographs of AuNP dispersions containing thrombin at different concentrations; (**B**) Absorption spectra of AuNP dispersions at various thrombin concentrations; (**C**) Calibration curve of thrombin using the absorbance ratio at 630 nm and 530 nm. Error bars indicate the standard deviation of the three measurements.

Next, it was investigated whether the color difference of the AuNP solution could be used to detect thrombin. As described above, the addition of thrombin induces double-strand formation between probes and synthesis of RNA strands that have a complementary sequence to the agDNA to inhibit the aggregation of ssDNA-AuNPs. Figure 3A shows the color of the AuNP solution after the addition of thrombin. As shown in the figure, there was a significant difference when higher concentrations of thrombin were added; the solution was red when more than 25 nM thrombin was added, while the color was blue upon the addition of thrombin at lower concentrations. The UV spectrum also supports a color difference with thrombin concentration (Figure 3B). As the thrombin concentration increased, the absorbance derived from the dispersed AuNPs increased, and the absorbance derived from the aggregated AuNPs decreased. The color difference due to AuNP aggregation can be evaluated by the absorbance ratio of A_630_/A_530_. A linear dependence of the A_630_/A_530_ value was observed for thrombin concentrations ranging from 3.1 to 25 nM. The limit of detection (LOD) of the proposed system was evaluated to be 4 nM. At this point the absorbance ratio was higher than the 3σ line, where σ denotes the standard deviation of zero-concentration background data. The LOD was similar to a result previously reported where thrombin was detected electrochemically using a binding-induced structural change in the aptamer [37]. The method proposed in this study does not rely on structural changes in aptamers, and it is therefore applicable to various sandwich methods using ligands such as antibodies and lectins.

### 3.5. Specificity of This Detection System

**Figure 4:**
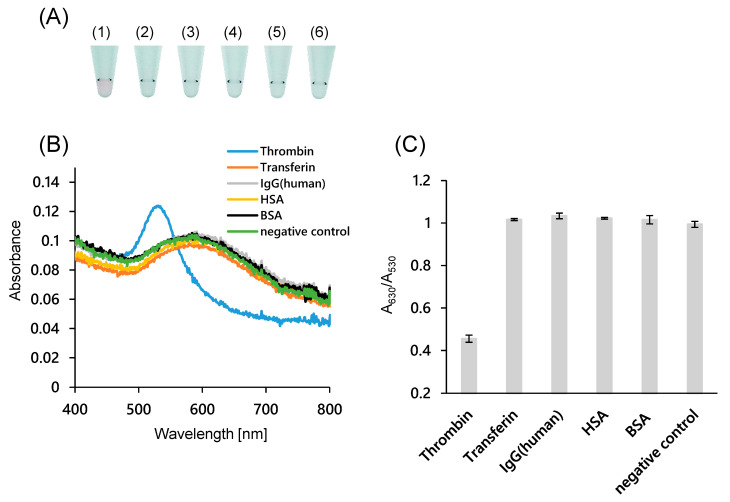
Specificity evaluation of colorimetric thrombin assay using 40 nm AuNPs modified with 15-bases DNA. (**A**) Photographs of the AuNP dispersions containing various proteins (100 nM). (1) α-thrombin, (2) transferrin, (3) IgG (Human), (4) HSA, (5) BSA, and (6) negative control (NA and NB were used instead of Probe-A and Probe-B); (**B**) Absorption spectra of AuNP dispersions with various proteins present; (**C**) Ratio of the absorbance at 630 and 530 nm in the presence of various proteins. Error bars indicate the standard deviation of the three measurements.

To evaluate the specificity of the system, several common proteins in human serum were added instead of thrombin. As shown in Figure 4A, the AuNP solutions were blue when 100 nM transferrin, human IgG, HSA, and BSA were added, while the solution was red when thrombin was added. The absorption spectrum (Figure 4B) and the absorbance ratio (Figure 4C) indicated that the AuNPs were dispersed only when thrombin was present. These results also suggest that this system is thrombin-specific and works in the presence of other proteins. This specificity is consistent with previous reports of thrombin detection using aptamers [38].

As another control experiment, 100 nM thrombin was added to the probe without the antithrombin aptamer sequence (NA and NB) (Figure 4). In the absence of aptamer sequences, AuNPs aggregated even when thrombin was added. This result indicates that the system works by a sandwich-type interaction between the two aptamer sequences and thrombin.

## 4. Conclusions

In this study, a novel amplified colorimetric method based on a transcription reaction for the detection of thrombin is presented. Signal amplification was achieved by a cyclic synthesis of RNA using the T7 polymerase. The polymerase reaction is triggered by the sandwich-type molecular recognition of the thrombin aptamers. In the presence of thrombin the AuNP solution appears red, and it is blue in its absence. The assay can discriminate thrombin from other common proteins with high selectivity.

In this study, we used the salt-aging method for the DNA modification of AuNPs. Recent studies showed other strategies for DNA immobilization [39,40]. For example, Ye et al. proposed a freeze-induced DNA modification of gold nanorods for a fast and dense DNA attachment [39]. Pei et al. proposed an immobilization of DNA on AuNPs using polyadenine as an anchoring block, which enables the controlled lateral spacing and surface density of DNA [40]. Interestingly, the high hybridization ability and rapid color change of the AuNP solution was obtained. It would be possible to obtain better sensing results by using these methods.

The advantage of this assay is that the target can be easily detected by the color differences of the AuNP solution. However, the transcription reaction for signal amplification is time consuming. It would be possible to reduce the transcription time by optimizing the reaction conditions, including addition of chemicals and peptides [41,42]. For example, the activity of T7 RNA polymerase can be enhanced several fold in the presence of an activator peptide [42]. It could also be possible to reduce the assay time by using an increased amount of reaction solution for incubation with AuNPs. In this case, however, buffer replacement would be necessary since components that may affect the dispersion state of AuNPs, such as DTT, are included in the current reaction solution.

As this method does not require structural changes in the aptamer, it can be easily adapted to molecular recognition mechanisms other than aptamers, such as biotin–streptavidin interaction, sugar recognition by lectins, and antigen–antibody reactions. The results presented in this study contribute to the construction of novel and versatile molecular detection systems with a variety of ligands.

## Supplementary Materials

The following are available online at https://www.mdpi.com/article/10.3390/s21134318/s1, Figure S1: Nucleotide sequence used in this experiment, Figure S2: Characterization of ssDNA-modified AuNPs, Figure S3: Fluorescence intensity change induced by hybridization of molecular beacon to RNA duplex and under different conditions.
