# Supplementary material for: Transcription-Based Amplified Colorimetric Thrombin Sensor Using Non-Crosslinking Aggregation of DNA-Modified Gold Nanoparticles
Source: Sensors (Basel). 2021 Jun 24;21(13):4318. doi: 10.3390/s21134318 (PMC8272040; doi:10.3390/s21134318)
Supplement: Supplementary file 1 [file sensors-21-04318-s001.zip › sensors-1255027-supplementary.pdf]

SUPPLEMENTARY MATERIAL

Transcription-Based Amplified Colorimetric Thrombin Sensor Using Non-Crosslinking Aggregation of DNA-Modified Gold Nanoparticles

Yu Muto [1,2], Gen Hirao [1] and Tamotsu Zako [1,*]

[1] Department of Chemistry and Biology, Graduate School of Science and Engineering, Ehime University, 2–5 Bunkyo, Matsuyama, Ehime, 790-8577, Japan; yuu-mutou-qr@tosoh.co.jp (Y.M.); f310165u@mails.cc.ehime-u.ac.jp (G.H.)

[2] Tokyo Research Center, TOSOH Corporation, 2743-1 Hayakawa, Ayase, Kanagawa 252-1123, Japan

[*] Correspondence: zako.tamotsu.us@ehime-u.ac.jp

Probe-A

5′-CGCCTAGGTTGGGTAGGGTGGTGGCGttttttttttAATTCTAATACGACTCACTATAGGGAGAGGCAATAGCTCTGGCAATTTCTTAggctag

Probe-B

5′-AGTCCGTGGTAGGGCAGGTTGGGGTGACTttttttttttCTTTTGCCGGTTGTTGACGATTCGTAGTTGATGATCGGGTActagcc

ssDNA-15

HS-$(CH_2)_6$-5′-TACCCGATCATCAAC

ssDNA-20

HS-$(CH_2)_6$-5′-TACCCGATCATCAACTACGA

agDNA-15

5′-GTTGATGATCGGGTA

agDNA-20

5′-TCGTAGTTGATGATCGGGTA

RNA-15

5′-UACCCGAUCAUCAAC

NA

5′-ttttttttttAATTCTAATACGACTCACTATAGGGAGAGGCAATAGCTCTGGCAATTTCTTAggctag

NB

5′-ttttttttttCTTTTGCCGGTTGTTGACGATTCGTAGTTGATGATCGGGTActagcc

MB

FAM-5′-GCCAGCTCGTAGTTGATGATCGGGTAGCTGGC-BHQ1

Figure S1 Nucleotide sequences of the DNA probes (Probe-A and Probe-B), the AuNP surface-bound thiolated DNA (ssDNA-15, ssDNA-20), complementary DNA (agDNA-15, agDNA-20), RNA (RNA-15), negative control probe (NA, NB), and MB. The underlined parts of Probe-A and Probe-B function as anti-α-thrombin DNA aptamers. For Probe-A and Probe-B, NA and NB, the small blue letters at the 3′-end indicate the complementary sequence for hybridization. For Probe-A and NA, T7-RNA promoter sequences were shown in red. For MB, FAM and BHQ1 indicate a fluorescent dye (6-carboxyfluorescein) and a quencher (Black Hole Quencher 1), respectively.

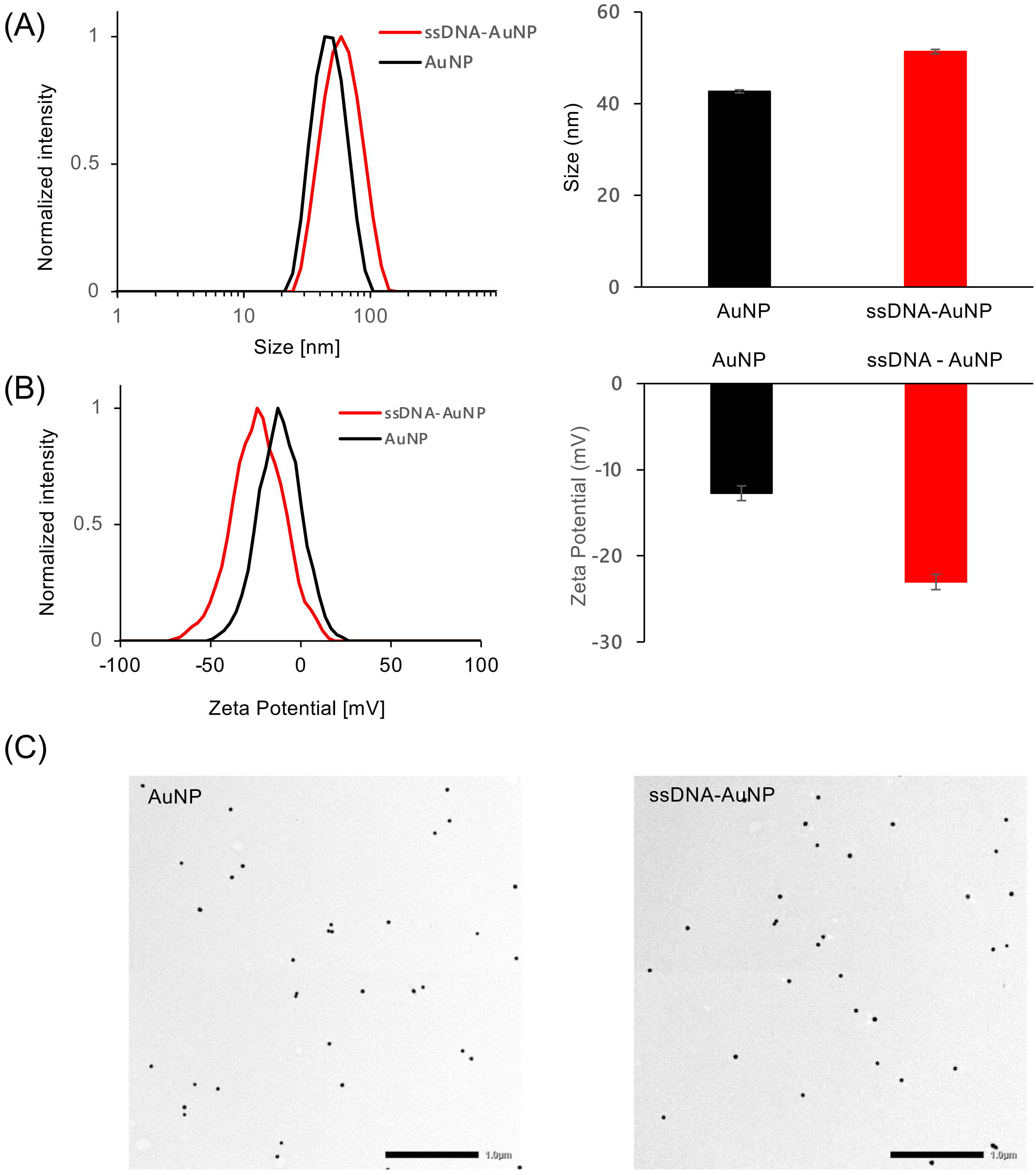

Figure S2 Characterization of ssDNA-modified AuNPs

(A) The size of 10 pM unmodified 40 nm AuNPs (AuNP) and ssDNA-modified 40 nm AuNPs (ssDNA-AuNP) was evaluated with dynamic light scattering analysis using Zetasizer-Nano ZS (Malvern Worcestershire, UK) (left). The average values of three measurement were shown in the right panel.

(B) The zeta potential values of 5 pM AuNPs and ssDNA-AuNP were measured with Zetasizer-Nano ZS (left). The average values of three measurement were shown in the right panel.

(C) TEM images of 40 nm AuNPs (left) and ssDNA-AuNPs (right). Transmission electron microscopy (TEM) was performed in order to confirm the dispersion of ssDNA-AuNPs. 10 µL sample was spotted on collodion-film coated TEM copper grids and allowed to adsorb. Samples were examined with an excitation voltage of 80 kV using a JEM-1400Plus TEM (JEOL, Tokyo, Japan).

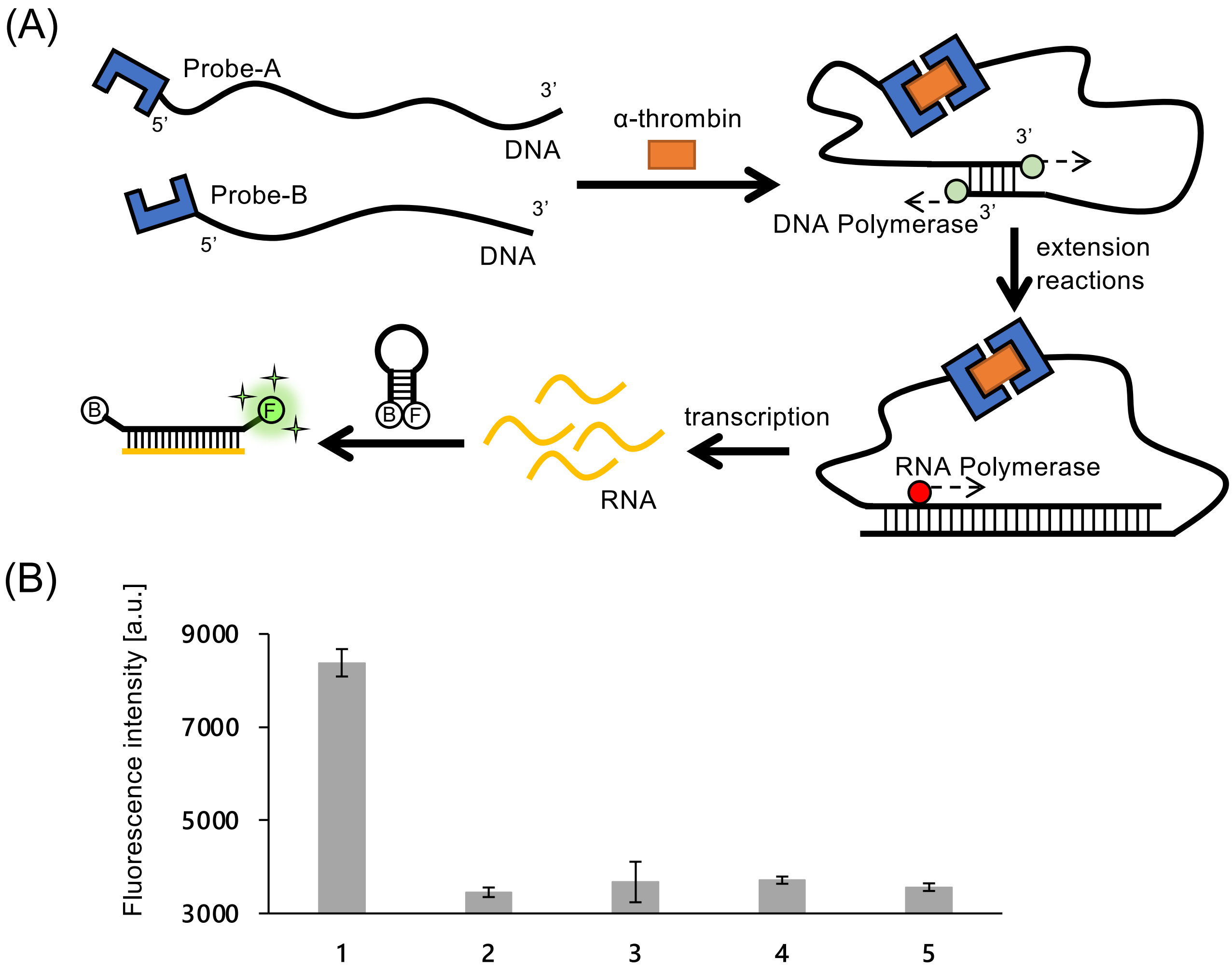

Figure S3 (A) Schematic illustration of the fluorescence intensity change induced by hybridization of MB to RNA duplex. (B) Fluorescence intensity changes of MB under different conditions: (1) 100 nM α-thrombin, (2) 0 nM α-thrombin, (3) without DNA polymerase, (4) without RNA polymerase, and (5) without aptamer units (NA and NB were used instead of Probe-A and Probe-B). Reaction temperature was 42℃.
